# Analyzing Liver Surface Indentation for *In Vivo* Refinement of Tumor Location in Minimally Invasive Surgery

**DOI:** 10.1007/s10439-020-02698-4

**Published:** 2020-11-30

**Authors:** Yingqiao Yang, Kai-Leung Yung, Tin Wai Robert Hung, Kai-Ming Yu

**Affiliations:** Department of Industrial and Systems Engineering, The Hong Kong Polytechnic University, R606, 1 Yuk Road, Hung Hom, Kowloon Hong Kong

**Keywords:** Soft tissue modeling, Inverse finite element analysis, Tumor locating, Surgical indentation, Robotic-assisted minimally invasive surgery

## Abstract

Manual palpation to update the position of subsurface tumor(s) is a normal practice in open surgery, but is not possible through the small incisions of minimally invasive surgery (MIS). This paper proposes a method that has the potential to use a simple constant-force indenter and the existing laparoscopic camera for tumor location refinement in MIS. The indenter floats with organ movement to generate a static surface deformation on the soft tissue, resolving problems of previous studies that require complicated measurement of force and displacement during indentation. By analyzing the deformation profile, we can intraoperatively update the tumor’s location in real-time. Indentation experiments were conducted on healthy and “diseased” porcine liver specimens to obtain the deformation surrounding the indenter site. An inverse finite element (FE) algorithm was developed to determine the optimal material parameters of the healthy liver tissue. With these parameters, a computational model of tumorous tissue was constructed to quantitatively evaluate the effects of the tumor location on the induced deformation. By relating the experimental data from the “diseased” liver specimen to the computational results, we estimated the radial distance between the tumor and the indenter, as well as the angular position of the tumor relative to the indenter.

**Electronic supplementary material:**

The online version of this article (10.1007/s10439-020-02698-4) contains supplementary material, which is available to authorized users.

## Introduction

Imaging techniques such as computed tomography (CT) or magnetic resonance imaging (MRI) allow clinicians to detect tumor(s) and map their position inside the human body before surgery. However, the location of a tumor at the intraoperative stage may have moved from where it was located due to living organs moving with body orientation, respiration, and surgical manipulations.^10^,^25^ Updating the intraoperative tumor location is necessary for surgeons to make precise resections with minimum damage to the organ. Although there are different types of tumors and some of them are softer than the surrounding tissue, in this study, we mainly focus on the tumors typically stiffer than the healthy tissue, which are also the majority.^14^,^28^ Surgeons in open surgery exploit this fact to update the tumor location intraoperatively as the stiffer tissue can be detected by finger sensation. However, with the advent of Minimally Invasive Surgery (MIS) and Robotic-assisted Minimally Invasive Surgery (RMIS), surgeons have to operate surgical instruments through small incisions, which restrict their sensation of palpation and increase the difficulty of precise tumor locating during surgery. This leads to a reliance on robotic palpation as a simple alternative to manual palpation.

Several robotic palpation systems have been developed to enable intraoperative tumor locating during MIS/RMIS. Indentation methods were frequently used to locate abnormalities based on measuring the force and displacement of the indenter during tool-tissue interaction (McCreery *et al.*^23^; Yamamoto *et al.*^37^; McKinley *et al.*^24^). Liu *et al.*^21^ proposed a rolling approach that moved a force-sensed roller over the tissue surface at a constant indentation depth to obtain a continuous stiffness distribution. Similar methods such as sweeping palpation (Ahn *et al.*^2^) and sliding palpation (Trejos *et al.*^35^) were also developed. One of the common points of these studies is that they rely on both palpation force and displacement to infer the tumor location. This method suffers as living organs move constantly during surgery, and hence it is not practical to maintain a precise indentation depth as in Liu *et al.*,^21^ or to obtain steady and accurate force and displacement measurement.

By using a constant-force indenter, which by definition is capable of floating up and down with the organ movement, a static indentation can be generated. The tumor location can be estimated by analyzing the static indentation profile captured by an existing laparoscope in MIS/RMIS, without the complexity of measuring the dynamic force and displacement of a moving target. A similar idea has been tested by Kawahara *et al.*^18^ In their work, an air jet was used to exert a constant force onto the soft tissue to produce a deformed surface, which was then captured by a camera. The stiffer object inside the soft tissue was located by analyzing the surface profile. However, their results were also affected by experimental conditions such as organ movement, which varies the distance between the tissue and the air jet and thus cannot be extended to more general applications. In addition, they also failed to give a quantitative estimation of the tumor location. Finite element (FE) modeling offers possible solutions for this problem. Sangpradit *et al.*^30^ and Ahn *et al.*^2^ developed FE models to locate abnormalities quantitatively. However, the former acquired the material parameters of the FE model by conducting uniaxial tests, which is not feasible in the scenario of MIS/RMIS; and the latter conducted experiments on silicone phantom and assumed the soft tissue had linear elasticity, which is far from the truth.

In this paper, we explored the use of a constant-force indenter irrespective of the organ movement, captured the indentation profile for analysis, and eventually constructed tumorous FE models to predict the exact tumor location in real-time. This constant-force indenter has the prospect of simple implementation by a weight at the end of a low friction cantilever design, allowing easy insertion through the trocars of MIS. The investigation started with indentation tests on healthy porcine liver specimens where the deformed surfaces were captured by a three dimensional (3D) scanner. By performing the inverse FE analysis and optimally matching computational deformation against the scanned image of the indentation, the material parameters of the healthy liver tissue were estimated. A pseudo-tumor was then put inside the healthy specimen to simulate the tumorous specimen, on which indentation tests were performed and corresponding indentation shapes were captured. Finally, computational models of tumorous tissue indentation were developed concurrently to predict the experimental deformation behavior for tumor locating. It should be noted that our method was intended for tumors stiffer than the healthy tissue, and their approximate locations have already been estimated before the operation. Whether this method will be used at the intraoperative stage is determined according to the diagnosis beforehand.

## Materials and Methods

### Indentation Test on Healthy Porcine Liver Tissue

Intact porcine livers were purchased from a local market in the early morning when they were just delivered from the central slaughterhouse and then transported to the laboratory within 15 minutes to ensure freshness. Indentation tests were conducted immediately on the fresh porcine liver specimens to simulate the procedure in MIS/RMIS. All tests were completed within 4 hours, and liver specimens were preserved in phosphate-buffered saline solution to minimize any possible dehydration.

**Figure 1:**
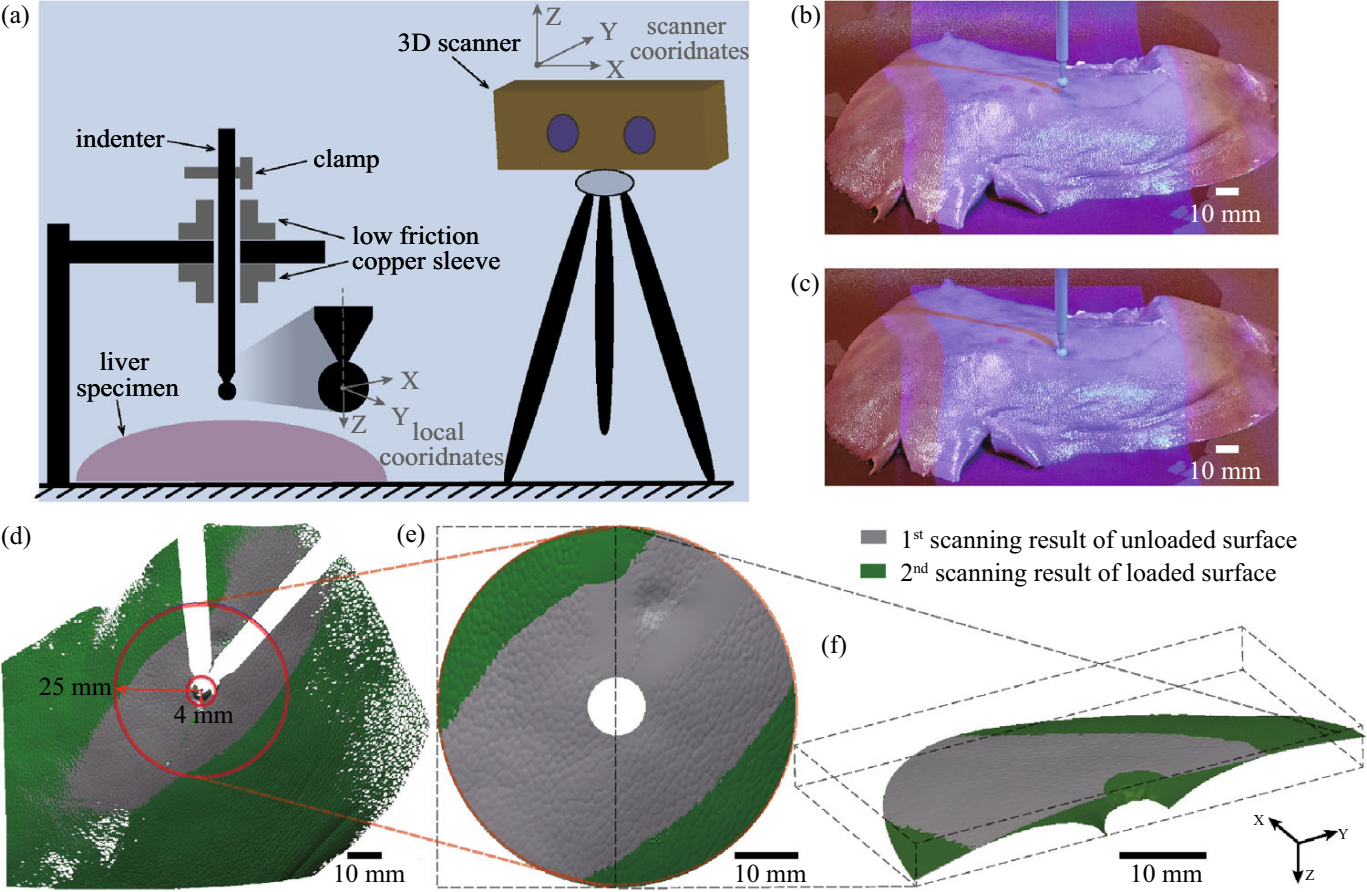
Indentation test on healthy porcine liver specimens and data acquisition. (a) The schematic of the experimental setup (an indenter with spherical tip of 6 mm in diameter was used to apply a constant force on the liver specimen by its mass of 60 g); (b) The reference scan made before the specimen was loaded; (c) the second scan made after the specimen was loaded; (d) Results of the two scans at the ROI; (e) Re-wrapped ROI from the top view; (f) 3D sectional view of ROI to show the two scanning results.

The experimental setup is shown in Fig. 1a. An indenter with 6 mm diameter spherical tip was used to indent the liver specimen. The indenter was 60 g and was held with low friction bearings giving negligible vertical friction so that a constant gravitational force of 0.588 N was exerted on the specimen. For the diameter and the mass of the indenter, we carried out ten times of measurements, and the actual values were 6.1 ± 0.01 mm, 60.2 ± 0.01 g by reporting the uncertainty. The weight and the tip diameter of the indenter were carefully selected to avoid tissue damage while having sufficient indentation.^9^ An optical scanner COMET L3D 5M (ZEISS OPTOTECHNIK, Germany) with structure light was employed to capture the 3D deformation of the tissue. It was equipped with a projector to generate blue light in a fringe pattern and a camera with a resolution of $2448 \times 2050$ pixels. The scanner was pre-calibrated to scan objects with a point resolution of 0.1 mm.

A scan was made to acquire the specimen’s initial profile in its undisturbed state as a reference, see Fig. 1b. After this reference scan, the indenter was released so that the specimen was loaded by the force of 0.588 N. The scanner then made the second scan to capture the specimen deformation after being loaded as in Fig. 1c. The original data in the scanner coordinate system was converted into a local coordinate system for data processing. The spherical center of the indenter tip in the reference scan defined the local origin, and the axis of the cylindrical body defined the local *z*-axis, Fig. 1a. All scanned meshes were pre-processed in the software Geomagic Design X (3D Systems, Inc., USA).

Indentation tests were performed on three specimens with thicknesses of $20 \pm 0.1$, $22 \pm 0.1$, and $25 \pm 0.1$ mm, obtained from ten times of thickness measurements for each case. For each specimen, three indentation tests were carried out at three selected positions around the central region of the specimen that had a relatively flat horizontal surface because the indenter depends on the gravitational force. Two scanning results of a representative specimen are shown in Fig. 1d: gray and green represent the reference and indented scans, respectively. The missing parts (in white) are caused by the occlusion of the surface by the indenter body and its shadow. Since the region far from the indenter tip has little deformation and the region underneath the indenter tip was occluded, the region of interest (ROI) was defined as between the two red circles in Fig. 1d, ranging from 4 to 25 mm. We further re-wrapped the missing parts and filled holes in the ROIs by using Geomagic Design X, as in Fig. 3c. Figure 3f presents a 3D sectional view of ROIs showing the two scanning results. After pre-processing, meshes of ROIs were exported as *.PLY format and loaded into MATLAB.

**Figure 2:**
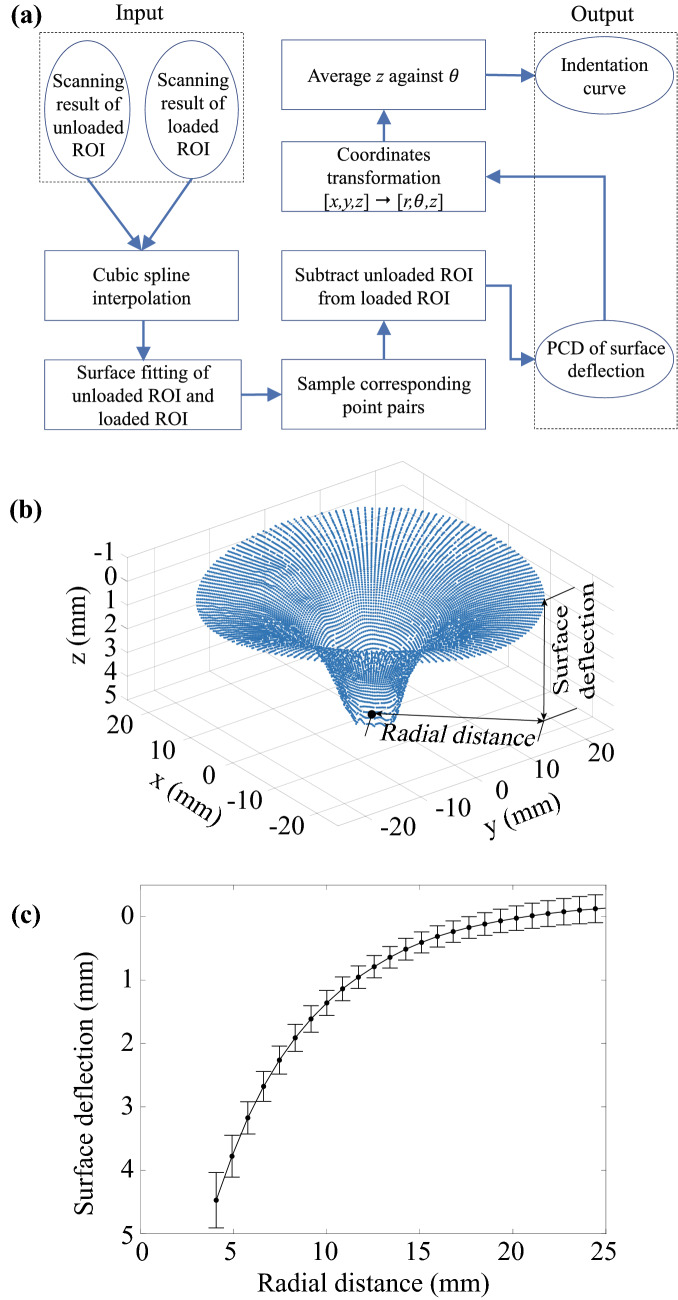
Data processing procedure and outputs. (a) The flowchart of data processing; (b) surface deflection profile of one indentation; (c) Averaged indentation curve from three indentations (mean and standard error).

The ROIs of unloaded and loaded surfaces were then processed to extract the deformation profile induced by the indentation for further analysis. The procedure of data processing is shown in Fig. 2a. A cubic spline interpolation was used with the ROIs to convert the discrete point cloud data (PCD) into continuous surfaces. The corresponding point pairs were sampled with the same *x* and *y* coordinates on the two surfaces. A Surface deflection profile was created by subtracting the points on the unloaded ROI surface from their counterparts on the loaded ROI surface, shown in Fig. 2b. After transforming data in Cartesian coordinates (*x*, *y*, *z*) into polar coordinates ($r, \theta , z$), slices of the surface were taken to create plots of *z* (the averaged perpendicular surface deflection of the three indentations) against *r* (the radial distance from the indenter tip), termed an indentation curve. Figure 2c shows the indentation curve (with mean and standard error) of a porcine liver specimen with a thickness of 22 mm.

### Inverse FE Method for Material Parameter Estimation

Abdominal organs such as the kidney, liver, and spleen are often considered to be isotropic, hyperelastic, and incompressible.^7^,^12^,^36^ The mechanical response of such materials is best described by using a strain energy function *U*, from which a material constitutive behavior is derived. The Ogden hyperelastic model^26^ has been widely used for solid soft tissue characterization, such as porcine liver tissue^13^,^20^ and brain tissue^4^. The strain energy function of the Ogden model defined in the Abaqus manual^8^ is:

1 $$\begin{aligned} U = \sum \limits _{i = 1}^N {\frac{{2{\mu _i}}}{{\alpha _i^2}}} ({\overline{\lambda }}_1^{ {\alpha _i}} + {\overline{\lambda }}_2^{ {\alpha _i}} + {\overline{\lambda }}_3^{ {\alpha _i}} - 3) + \sum \limits _{i = 1}^N {\frac{1}{{{D_i}}}} {({J_{el}} - 1)^{2i}} \end{aligned}$$

where $\mu _{i}$ and $\alpha _{i}$ are material constants, and *i* is the number of terms; $\lambda _{1}$, $\lambda _{2}$, and $\lambda _{3}$ are principal stretches in three directions. $D_{i}$ and $J_{el}$ are temperature-dependent variables, and $J_{el}$ is equal to 1 for incompressible material. The first order Ogden model ($i = 1$) was used as the constitutive equation to simplify the analysis. It uses only the parameters $\mu$ and $\alpha$, as in (2).

2 $$\begin{aligned} U = \frac{{2\mu }}{{\alpha _{}^2}}({\overline{\lambda }} _1^{\alpha } + {\overline{\lambda }} _2^{\alpha } + {\overline{\lambda }} _3^{\alpha } - 3) \end{aligned}$$

For unconfined uniaxial compression or tension, let $\lambda _3 = \lambda$ be the principal stretch in the load direction. By assuming a homogeneous deformation, we have $\lambda _3 = {1}/{{\lambda _{1}}^2} ={1}/{{\lambda _{2}}^2}$. The nominal stress *T* of the first order Ogden model under the uniaxial test in Abaqus manual^8^ is given by

3 $$\begin{aligned} T = \frac{{2\mu }}{{\alpha _{}}}(\lambda ^{\alpha -1} - \lambda ^{-\frac{1}{2}\alpha -1}) \end{aligned}$$

**Figure 3:**
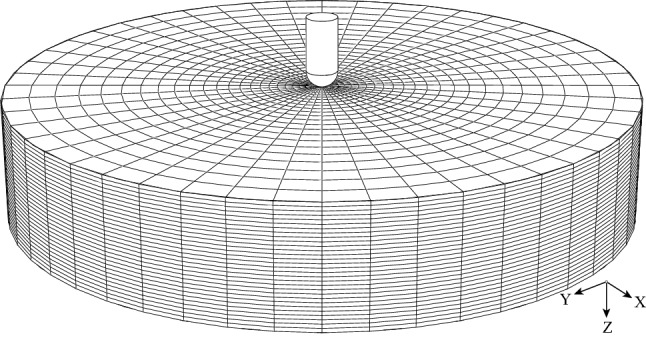
The FE model of the healthy liver tissue indentation in the reference configuration. The bottom of the model was fully constrained. An indentation force of 0.588 N was applied on the indenter to simulate the indentation test.

FE modeling of the indentation test was conducted using Abaqus (Version 6.14-3). As shown in Fig. 3, the indenter was modeled as an analytical rigid body with a hemispherical bottom and 6 mm in diameter. The liver specimen was modeled as a deformable cylinder with 22 mm in height and 120 mm in diameter. The liver model was meshed with $33\,120$ hexahedral C3D8RH elements and 1440 wedge C3D6RH elements. Convergence tests were performed to define an appropriate mesh size. The contact between the indenter and the tissue was defined as a frictionless surface-to-surface contact. During the tool-tissue interaction, the bottom face of the liver model was fully constrained. Since the porcine liver is very soft, it is deformed by gravity, causing non-zero internal stress. In the computational simulation, a gravitational force was first exerted on the liver model to simulate the tissue’s natural deformation. Subsequently, the force load of 0.588 N was imposed on the indenter to induce an indentation on the tissue.

The indentation curve was extracted from the modeling results according to the flowchart in Fig. 2a, and then an inverse FE method was developed to determine the optimal material parameters $\mu$ and $\alpha$ in (2). A Genetic Algorithm (GA) was employed for optimization because of its ability to handle discontinuous objective function and find global minima for highly nonlinear problems. Similar GA-based parameter estimation methods for soft tissue biomechanics studies can be found in previous work.^11^,^15^

**Figure 4:**
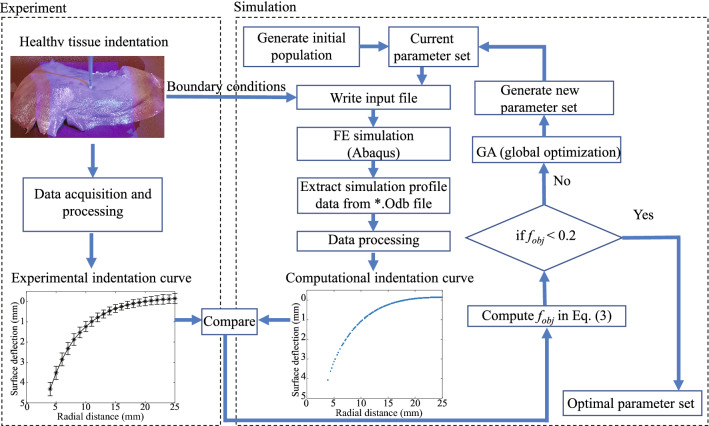
The flowchart of GA-based inverse FE optimization to acquire the material parameters of the porcine liver tissue. Varied material parameters were written into the FE model in each iteration, until the computational indentation curve was closed enough with the experimental indentation curve.

Figure 4 shows the flowchart of the GA-based inverse FE optimization. In the beginning, an initial population was generated with 20 random individuals, and each individual was associated with a parameter set of the constitutive model. The parameter set was then written into an Abaqus input file for FE modeling to simulate the tissue deformation under the indentation load of 0.588 N. An indentation curve was extracted from the FE model for comparison with the experimental data. The distance between the two curves was defined as the objective function, given by

4 $$\begin{aligned} f_{obj}(\mu ,\alpha ) = \sqrt{\frac{1}{n}\sum \nolimits _{i = 1}^n {{{({S_{FE}^i} - {S_{EXP}^i})}^2}} }, \end{aligned}$$

where $n = 80$ is the number of sample points on the two curves, $S_{FE}^i$ is the predicted surface deflection at sample point *i* on the computational indentation curve, and $S_{EXP}^i$ is the real surface deflection at sample point *i* on the experimental indentation curve. The objective function is minimized by evolutionarily updating the parameter set. An optimal material parameter set is obtained when $f_{obj}(\mu ,\alpha )$ is less than 0.2, meaning the computational indentation curve is close enough to the experimental one. The GA was performed by using a Matlab built-in library. A toolbox^27^ that connects Abaqus with Matlab was employed to automatically extract the 3D coordinates of nodes from Abaqus output database files.

### Tumor Locating

**Figure 5:**
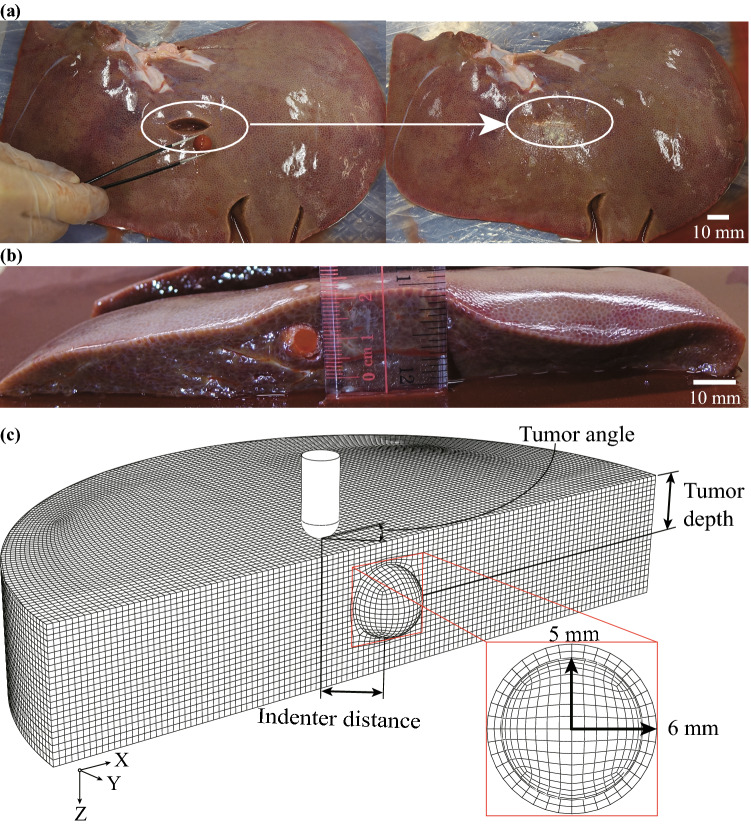
Indentation test and FE model of tumorous tissue. (a) Tumorous specimen preparation, a silicone ball in 10 mm diameter was glued into the same healthy porcine liver specimen to serve as a tumor; (b) measurement of the tumor depth; (c) the FE model of the tumorous liver tissue indentation (only half-model shown) in the reference configuration. A layer of the glue was assumed to be in a spherical shape surrounding the pseudo-tumor.

After conducting three indentation tests on the healthy specimen, we immediately prepared it as a “diseased” specimen to prevent decay. This ensured that the tumorous specimen would have the same fundamental properties as the healthy specimen. Figure 5a shows the preparation of the cancerous specimen: a small incision was cut from the bottom of the healthy tissue, and then a silicone ball with 10 mm in diameter was put inside it as a pseudo-tumor. In real life, a mature tumor is often surrounded by immature tumorous tissue. The ball was wrapped with a thin layer (around 1 mm in thickness) of transglutaminase, also called “meat glue,” in advance to represent the immature tumorous tissue and to bond to the specimen. The Young’s modulus of the ball is 1 MPa, which is similar to the mean modulus of the diseased liver (0.74 MPa) reported by Carter *et al.*^5^. The incision was then sealed by the “meat glue”.

After finishing the preparation, the cancerous specimen was turned over, and the indentation tests were conducted from the top of the tissue. We marked the position of the tumor and carried out indentation tests with indenter distance (defined in Fig. 5c: the radial distance between the center of the tumor and the indenter tip) of 0 mm, 10 mm, and 20 mm, respectively. For each indenter distance, three indentations were performed. The PCD of the surface deflection and averaged indentation curve were acquired for each case according to the data processing procedure in Fig. 2a. After finishing all tests, we cut the specimen through the silicone ball to measure the tumor depth (defined in Fig. 5c: the vertical distance between the center of the tumor and the top surface of the specimen). As shown in Fig. 5b, the tumor depth is 10 mm.

The geometry of the tumorous tissue model was similar to that of healthy tissue in Fig. 3, except a tumor was embedded inside. As shown in Fig. 5c, the tumor is modeled as a spherical body with 10 mm in diameter and at a 10 mm depth, to match the experiments. The thin layer of glue surrounding the ball was modeled in a spherical shape with a thickness of 1 mm. The material properties of the tumor tissue were set to be the same with the silicone ball, with a Young’s modulus of 1 MPa and a Poisson’s ratio of 0.45. The Young’s modulus of the immature tumorous tissue was assumed to be 0.34 MPa empirically, softer than the mature tumorous tissue and stiffer than the healthy tissue. For more accurate cancerous tissue modeling, a future study should focus on the characterization of cancerous tissue properties and tumor growth, which is beyond the scope of this study. The first order Ogden model with the optimal material parameters obtained in Sect. 2.2 was used for the healthy part of liver tissue in the FE model. In Fig. 5c, the tumor angle is defined as the angular position of the radial line passing through the center of the tumor and the indenter tip with respect to the *x*-axis. The model was meshed with 308,829 hexahedral C3D8RH elements. Convergence tests were performed to determine an appropriate mesh size.

In the computational simulation, a gravitational force was exerted on the model first, and then the indentation force of 0.588 N was imposed on the indenter to induce an indentation on the tissue. Five indentation tests were carried out on the tumorous tissue model with indenter distances of 0, 5, 10, 15, and 20 mm. It is noted that the tissue thickness and tumor depth change slightly when the tissue is subjected to the gravitational force, but the variation is too small to be considered significant.

As the tumor location in the computational model was a known quantity, the modeling results of the tumorous FE model with varied indenter distances were used as templates for assessing the experimental results. The unknown tumor location in experiments can be estimated by matching the experimental data with the computational templates. Let template 1, template 2, template 3, template 4, and template 5 denote the computational indentation curves extracted from tumorous FE models with indenter distances of 0, 5, 10, 15, and 20 mm, respectively. Template 6 is the computational indentation curve extracted from the FE model of healthy tissue. The experimental indenter distance was estimated by comparing the experimental indentation curve with the six templates in turn to determine the most similar template. The similarity was estimated by calculating the distance between the experimental indentation curve and the template by using (4).

In the previous step, the PCD of the surface deflection (see Fig. 2b) was acquired from experiments and FE models. The experimental PCD was aligned with the computational PCD to determine the experimental tumor angle. The computational PCD was set as the target, and the experimental PCD was rotated to match the target during an Iterative Closest Point (ICP) procedure until the minimum Euclidean distance was achieved. A Delaunay triangulation was used to acquire a more accurate registration result and improve computational efficiency. It should be noted that no translation was applied to the experimental data as these two sets of data were already pre-aligned. After ICP, a rotation matrix was obtained, and the rotation angle can be derived as the tumor angle.

## Results

### Identified Tissue Material Parameters

**Figure 6:**
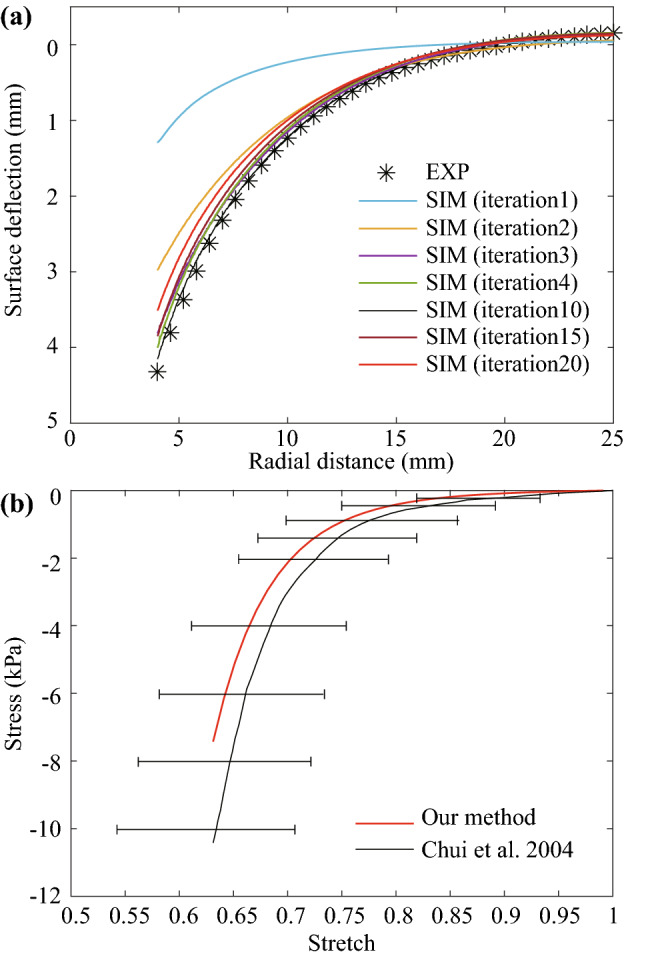
The optimization result and verification of the optimal material parameters. (a) comparison of iteratively generated computational indentation curve and experimental data; (b) comparison of the analytical stretch-stress curve calculated based on the optimal parameters with the experimental stress-stretch curve of porcine liver tissue in the work of Chui *et al.*^7^

The optimization result is partly shown in Fig. 6a, where the computational indentation curve generated from the healthy FE model gradually approaches the experimental data. The inverse FE analysis was carried out ten times, and the material parameter set with the smallest value of $f_{obj}(\mu ,\alpha )$ was selected as the optimal one. The result of the inverse FE analysis was the values for the material parameters of $\mu = 169.34~\mathrm {Pa}$ and $\alpha = -11.78$, with a corresponding objective function value of 0.12. Such material parameters also coincide with the results of our previous work^38^ on *ex vivo* porcine liver tissue characterization, where $\mu = 170~\mathrm {Pa}$ and $\alpha = -12.25$.

To further verify the optimal material parameters, we used $\mu = 169.34~\mathrm {Pa}$ and $\alpha = -11.78$ in (3) to acquire the analytical stress-stretch curve that is commonly used to reveal properties of a material. The experimental stress-stretch curve acquired by Chui *et al.*^7^ was plotted for comparison, see Fig. 6b. In their work, they conducted uniaxial compression tests on 70 samples from 20 livers to acquire the stress-stretch curve of porcine liver tissue. Noted that we only compared the compression here since indentation mainly involves compression mode according to our previous experimental and computational results.^38^

### Tumor Locating

**Figure 7:**
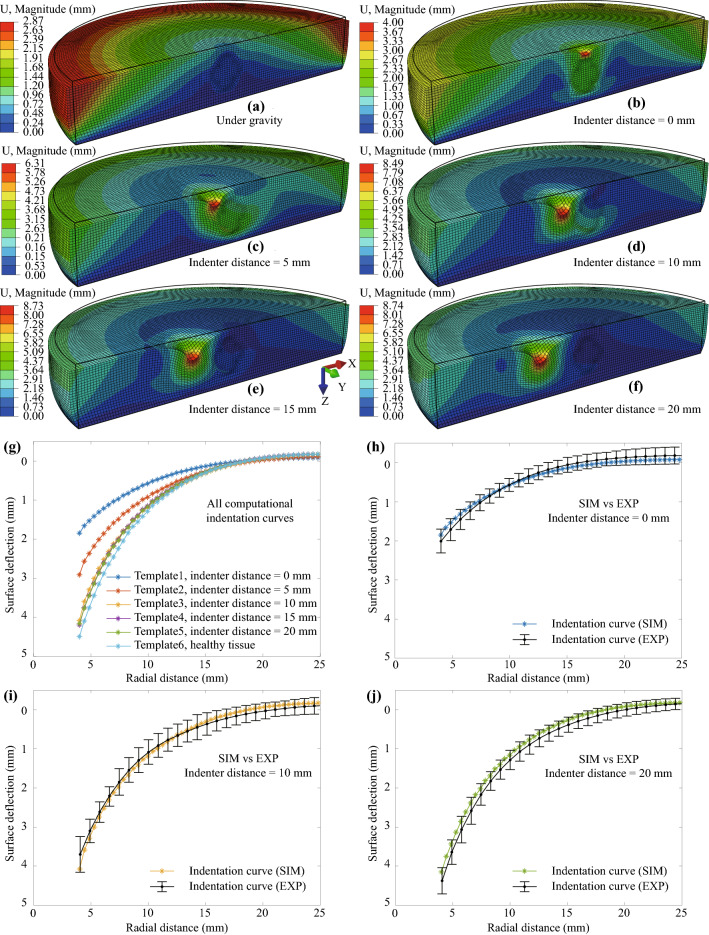
Computational results of tumorous tissue indentation and comparison with experimental data. Displacement distribution on the tumorous tissue: (a) under gravitational force only; Under gravity and indentation force of 0.588 N, with an indenter distance of (b) 0 mm, (c) 5 mm, (d) 10 mm, (e) 15 mm, (f) 20 mm (Black frames indicate the reference configurations of the models. For clarity, the indenter is not shown in plots). (g) indentation curves extracted from FE models. Comparison of computational results with experimental data: indentation curves acquired with an indenter distance of (h) 0 mm, (i) 10 mm, (j) 20 mm. Note, the corresponding maximum principal Catchy stress distribution of (a)–(f) can be found in the electronic supplement to this article.

The FE modeling results of the tumorous tissue indentation are given in Fig. 7. Figure 7a shows the displacement distribution on the tumorous tissue under gravitational force only. The black frame indicates the initial configuration of the model. Figures 7b–7f show the corresponding results with an indenter distance of 0, 5, 10, 15, and 20 mm, respectively. The computational indentation curve of each case was extracted from the computational results as templates, see Fig. 7g. To evaluate the modeling accuracy, we compared the computational indentation curve with the corresponding experimental data. The results are given in Figs. 7h–7j, and the good agreement indicates that the tumorous FE model is good enough to predict the experimental deformation.

**Figure 8:**
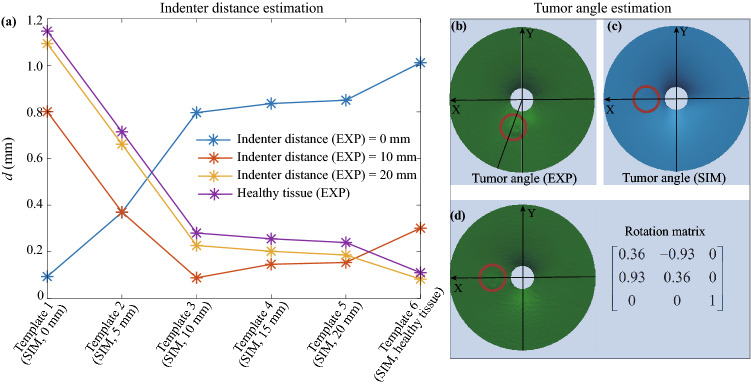
Indenter distance and tumor angle estimation. (a) Indenter distance estimation, the smaller the value in *d* means the higher similarity between computational and experimental data. Template 1–Template 6 are corresponding to the six computational indentation curves in Fig. 7g; (b) the triangulation result of the PCD from the experimental data with indication of the tumor angle (indenter distance = 10 mm); (c) the triangulation result of the PCD from the FE model (indenter distance = 10 mm); (d) the experimental result after ICP and the acquired rotation matrix.

Let the four experimental indentation curves serve as unknown testing data sets, and the six computational indentation curves are templates. The similarities between unknown data sets and each template are evaluated, as shown in Fig. 8a: the smaller the value of *d* means the higher similarity, and based on which the indenter distance can be estimated. For the “diseased” specimen, when the experimental indenter distance is 0 mm, its indentation curve is closest to template 1 (the indentation curve extracted from the FE model of tumorous tissue with a computational indenter distance of 0 mm) and farthest away from the template 6 (the indentation curve extracted from the FE model of healthy tissue). However, when it comes to the healthy specimen, the case is opposite, indicating that normal and abnormal tissue can be distinguished. From each polyline in Fig. 8a, by finding out the minimum value of *d*, we can approximately estimate the experimental indenter distance. Nevertheless, for the case with an experimental indenter distance of 20 mm, the pattern of the polyline is very similar to that of normal tissue (see the yellow and purple lines in Fig. 8a), which means that it might be difficult to discriminate healthy tissue from tumorous tissue under such a circumstance.

With the known indenter distance, the ICP algorithm was applied to align the experimental PCD to their computational counterparts to estimate tumor angle. With an indenter distance of 10 mm, the PCD of surface deflection extracted from the experiment and the FE model were triangulated as in Figs. 8b and 8c. The red circles indicate the tumor location. After applying ICP, Fig. 8d shows the aligned experimental results and the rotation matrix. A rotation angle, $68^{\circ }$, was derived from the rotation matrix, and its opposite value, $-68^{\circ }$, was the tumor angle in experimental data.

In addition, we also aligned the experimental PCD with the PCD from other FE models with indenter distances of 5 and 15 mm, and similar rotation angles were acquired. This implies that it is not necessary to know the exact indenter distance for tumor angle estimation.

## Discussion

In this study, by investigating the surface deflection from porcine liver specimens and FE models of the specimens, the material parameters of porcine liver tissue were estimated. The objective was to determine the location of a pseudo-tumor inside the liver specimen.

The proposed method for tissue parameter estimation can be effectively applied to an intact organ *in vivo* since only visual information and a single indentation are required. The indentation with an easily implemented constant force does not involve any sophisticated sensors to measure the force or displacement dynamically as in the studies (see, e.g., Ahn *et al.*^1^; Lister *et al.*^20^). The characterized parameters may change according to liver conditions (degree of freshness, degree of dehydration, whether the specimen experienced freeze-thaw processing, whether the stress-free state was carefully calibrated), as well as the experimental setup. The details about porcine liver tissue properties characterization can be found in previous work.^6^,^7^,^13^,^36^,^38^ According to our literature survey, the porcine liver stated in the work of Chui *et al.*^7^ is most similar to ours. Despite our result in Fig. 6b seeming to be slightly softer in material, it is within the range reported by Chui *et al.*^7^ The slight discrepancy might be caused by the different experimental conditions and variations of the tissue. It is concluded that the optimal material parameters are reasonable estimates of the properties of the porcine liver tissue. Compared with the previous work,^7^,^30^,^38^ where small samples were cut off from the organ to measure the tissue properties *ex vivo* by using uniaxial tests, the method mentioned in this work is more feasible for *in vivo* implementation. Since the soft tissue properties vary a lot between *ex vivo* and *in vivo* states,^20^ this *in vivo* soft tissue properties characterization method has significant clinical potential. There are also other related studies.^3^,^5^,^36^,^39^ on tissue characterization by measuring the force-displacement curve using the indentation method. However, given that abdominal organs are very soft and keep moving during surgery, it is not easy to measure precise indentation depth and reaction force simultaneously. Our method overcame this problem by using a constant-force indenter implementable by a simple weight combined with the possible visual observations of an optically calibrated laparoscopic camera in MIS. Noted that we used element typeI (hexahedral C3D8RH and wedge C3D6RH) for healthy tissue modeling in Fig. 3, and element typeII (Only hexahedral C3D8RH) for cancerous tissue modeling in Fig. 7. The reason is that by using typeI for healthy tissue, the procedure of determining the optimal material parameters can be accelerated. We constructed healthy FE models with these two element types, inputted the same optimal material parameters, and compared their corresponding indentation curves. The results (found in the electronic supplement to this article) suggested that the differences caused by element types on the indentation curve were not significant.

Furthermore, the work on quantitative tumor location estimation contributes to estimating the radial distance between the indenter tip and the tumor and determining the tumor angle relative to the indenter during the indentation procedure. These two indices can enhance surgeons’ intraoperative navigation for tumor locating and precise tumor resection during MIS/RMIS. In a practical application, before surgeons start to locate the tumor, an appropriate computational model of the tumorous tissue could be constructed according to prior knowledge such as data from MRI scans: tumor size, tumor depth, and tissue thickness, etc. Then, the computational model can be used to generate templates for a variety of indentations for the computer to match deformations captured from surgical sites. In this way, the tumor location can be estimated and displayed in front of the surgeons to assist their work. Although we did not implement the surface reconstruction and optimization algorithms in real-time, it does not affect the conclusion that our method can be used in real-time since the computation is implemented by analyzing a set of images captured by the optical device to return the optimal parameters and the prediction of tumor location. In recent studies.^19^,^33^, the computation-intensive algorithm in stereoscopic techniques has been optimized and distributed to many parallel processors provided by the graphics processing unit (GPU) to complete the computation within an acceptably short time. Some studies.^17^,^34^ focus on FE modeling of soft tissue deformation in real-time based on parallel computation. In our current application, instead of reconstructing all abdominal scenarios in real-time continuously, we implemented the surface reconstruction and optimization discretely, picture by picture. That is, we captured images of indentation deformation first and then converted them into point cloud data for analysis. To give an example of the computation time required, our analysis took 3–5 h with current hardware (a 3.6 GHz Intel Core i7-4790 machine) with a speed of 21.48 gigaFLOPS. If the same calculation were beamed through a high-speed network to be performed on a supercomputer such as Titan with a speed of 17.95 petaFLOPS in 2013 (almost $10^6$ times faster than our current hardware), we would obtain the result in less than 0.03 seconds. Given the current advancement of hardware, the computation time would be even shorter. Therefore, while the research could benefit from computation acceleration algorithms, the implementation of the current method is not constrained by computation time.

Despite that a 3D scanner was employed for data acquisition in this study, given the fact that vision techniques are quite mature in current RMIS systems, it will not be difficult to acquire a similar surface profile *in vivo* by using an optically pre-calibrated laparoscopic camera commonly found in MIS/RMIS operations.^32^ Using a 3D scanner enables us to focus on more fundamental issues in MIS. Previous studies.^20^,^37^ also used a 3D scanner as a starting point to acquire surface deformation for applications in MIS. The measurement based on a laparoscopic camera can be addressed by many other recent studies.^19^,^31^–^33^ In real applications of 3D deformation reconstruction during tool-tissue interaction in MIS, possible challenges exist, such as specular reflections, instrument occlusions, lack of features, etc. A comprehensive literature survey^22^ has summarized the recent optical 3D reconstruction techniques in MIS and identified the major technical challenges and future perspectives of the field. To solve the image distortion problem that occurred during the reconstruction procedure, Shahidi *et al.*^31^ have reported an automatic calibration routine to determine the intrinsic and extrinsic parameters of a laparoscopic camera and correct its lens distortion, where adequate accuracy has been achieved. This study focuses on investigating the theory behind the intraoperative tumor locating by demonstrating a constant-force indentation method combined with visual information. Further work in practical implementation would involve the optical dimension calibration of the laparoscopic camera in order to put the theory into practical use, but the objectives remain. The constant-force indenter can be designed with a simple weighted cantilever small enough to be inserted through the trocars into the abdominal cavity. The simple mechanical cantilever design and its corresponding clinical trial data will be the subject of another publication.

This method though convenient also has several limitations. For tissue modeling, the limitation is that the constitutive model did not involve shear mode related terms, or time-dependent effects caused by the viscoelastic behavior of liver tissue, which can be beneficial to an accurate characterization of the tissue properties. However, for the purpose of tumor locating, where the emphasis is on distinguishing between healthy tissue and tumorous tissue rather than investigating the details of tissue properties, the current approach would be acceptable. Most previous studies^2^,^18^,^21^ on a similar topic were carried out without involving the characteristics mentioned above. Moreover, we applied a gravitational force on the liver tissue before indentation to simulate the original state of liver tissue with pre-existing residual stress. There could always be further considerations in residual stresses for more accurate simulation, but that would have fallen into residual stress characterization, which is beyond the scope of this paper. According to our results, the indentation curves from simulation models match with experimental data well, indicating the current simplification of the FE model is acceptable. In addition, it is known that there is a thin capsule surrounding the liver, which may affect the mechanical response of liver tissue. Previous studies have investigated the effects of bovine liver capsule^16^ and porcine liver capsules.^20^ Future computational models could solve this problem by including such a capsule for more accurate simulation.

In terms of limitations on tumor locating, the tumor depth, size, and stiffness remained unchanged in this study since they are intended to be known at the preoperative stage. Nevertheless, future studies considering the effects of these values on the indentation curve will provide more insights for clinical applications. Besides, in our current work, we mainly indented the liver’s central part at a relatively flat horizontal surface to analyze the proposed method in a normalized manner with the indenter perpendicular to the tissue surface. For other locations, especially those near to the edge, the gravitational effects of the indenter and the tissue are the same, where the only difference will be the inclination angle of the organ surface that has to be accounted for in the corresponding FE model. Furthermore, instead of using sample cylindrical geometry to model liver tissue, FE modeling involving the real geometry of the liver specimen (see, e.g., Lister *et al.*^20^; Plantefève *et al.*^29^) and considering different boundary conditions and indenter shapes (see, e.g., Ahn *et al.*^1^) will be investigated in future work. The low specimen number could be another limitation, but we focus on investigating the theory behind the intraoperative tumor locating in this study. From a technical point of view, this study established an important starting point for research in this area. Future work will include extensive clinical trials to evaluate the effectiveness of this technique.

## Electronic supplementary material

Below is the link to the electronic supplementary material.

Electronic supplementary material 1 (PDF 10753 kb)
