# Supplementary material for: Analyzing Liver Surface Indentation for In Vivo Refinement of Tumor Location in Minimally Invasive Surgery
Source: Ann Biomed Eng. 2020 Nov 30;49(5):1402–15. doi: 10.1007/s10439-020-02698-4 (PMC8058013; doi:10.1007/s10439-020-02698-4)
Supplement: Supplementary file 1 — Electronic supplementary material 1 (PDF 10753 kb) [file 10439_2020_2698_MOESM1_ESM.pdf]

S1-Figure

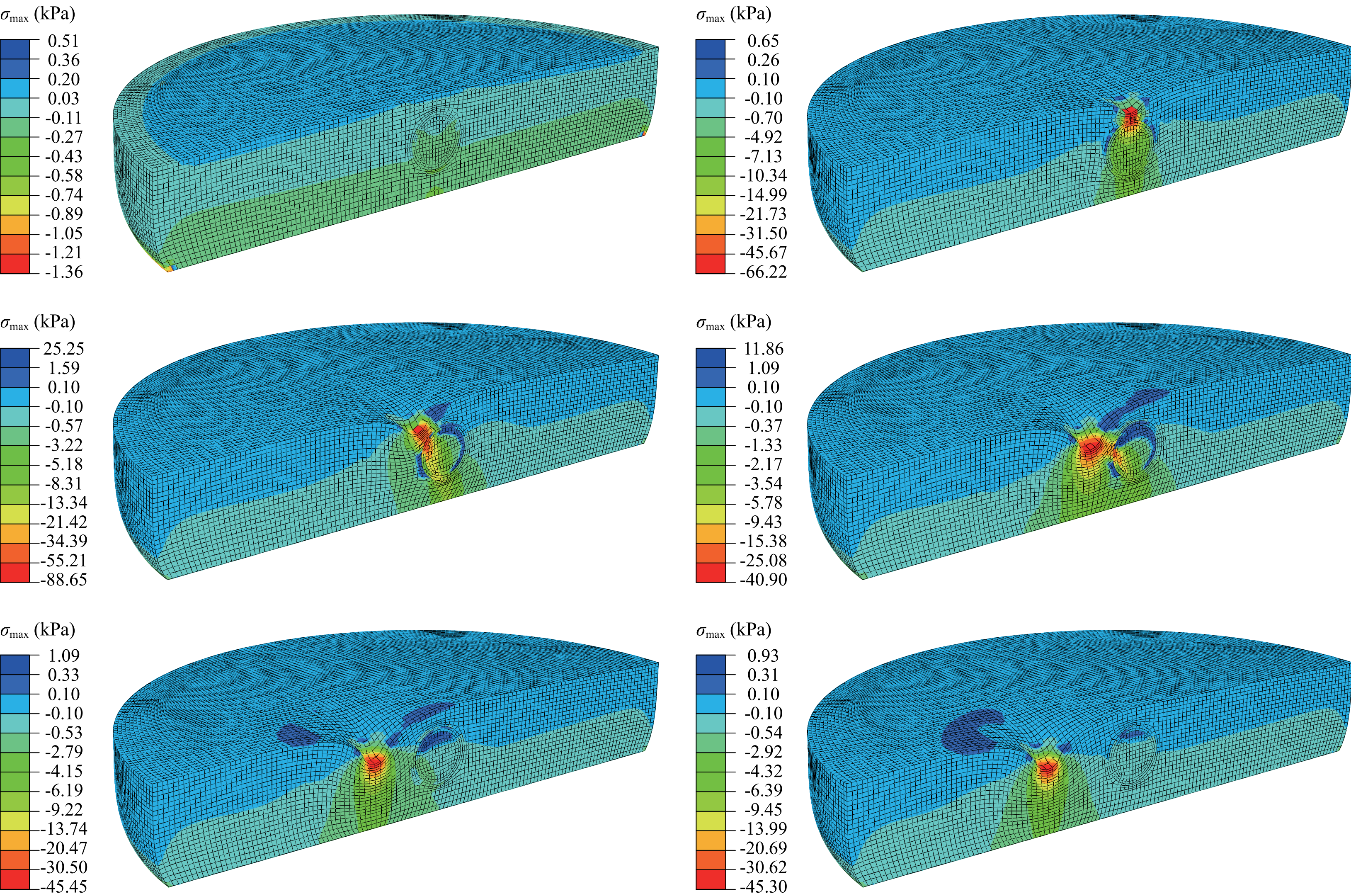
σmax (kPa)
0.51
0.36
0.20
0.03
-0.11
-0.27
-0.43
-0.58
-0.74
-0.89
-1.05
-1.21
-1.36
σmax (kPa)
0.65
0.26
0.10
-0.10
-0.70
-4.92
-7.13
-10.34
-14.99
-21.73
-31.50
-45.67
-66.22
σmax (kPa)
25.25
1.59
0.10
-0.10
-0.57
-3.22
-5.18
-8.31
-13.34
-21.42
-34.39
-55.21
-88.65
σmax (kPa)
11.86
1.09
0.10
-0.10
-0.37
-1.33
-2.17
-3.54
-5.78
-9.43
-15.38
-25.08
-40.90
σmax (kPa)
1.09
0.33
0.10
-0.10
-0.53
-2.79
-4.15
-6.19
-9.22
-13.74
-20.47
-30.50
-45.45
σmax (kPa)
0.93
0.31
0.10
-0.10
-0.54
-2.92
-4.32
-6.39
-9.45
-13.99
-20.69
-30.62
-45.30

Figure S1: *Maximum principal Cauchy stress distribution (in kPa) on the tumorous tissue with a tumor depth of 10 mm: (a) Under gravitational force only; Under gravity and indentation force of 0.588 N, with an indenter distance of (b) 0 mm, (c) 5 mm, (d) 10 mm, (e) 15 mm, (f) 20 mm.*

S2-Figure

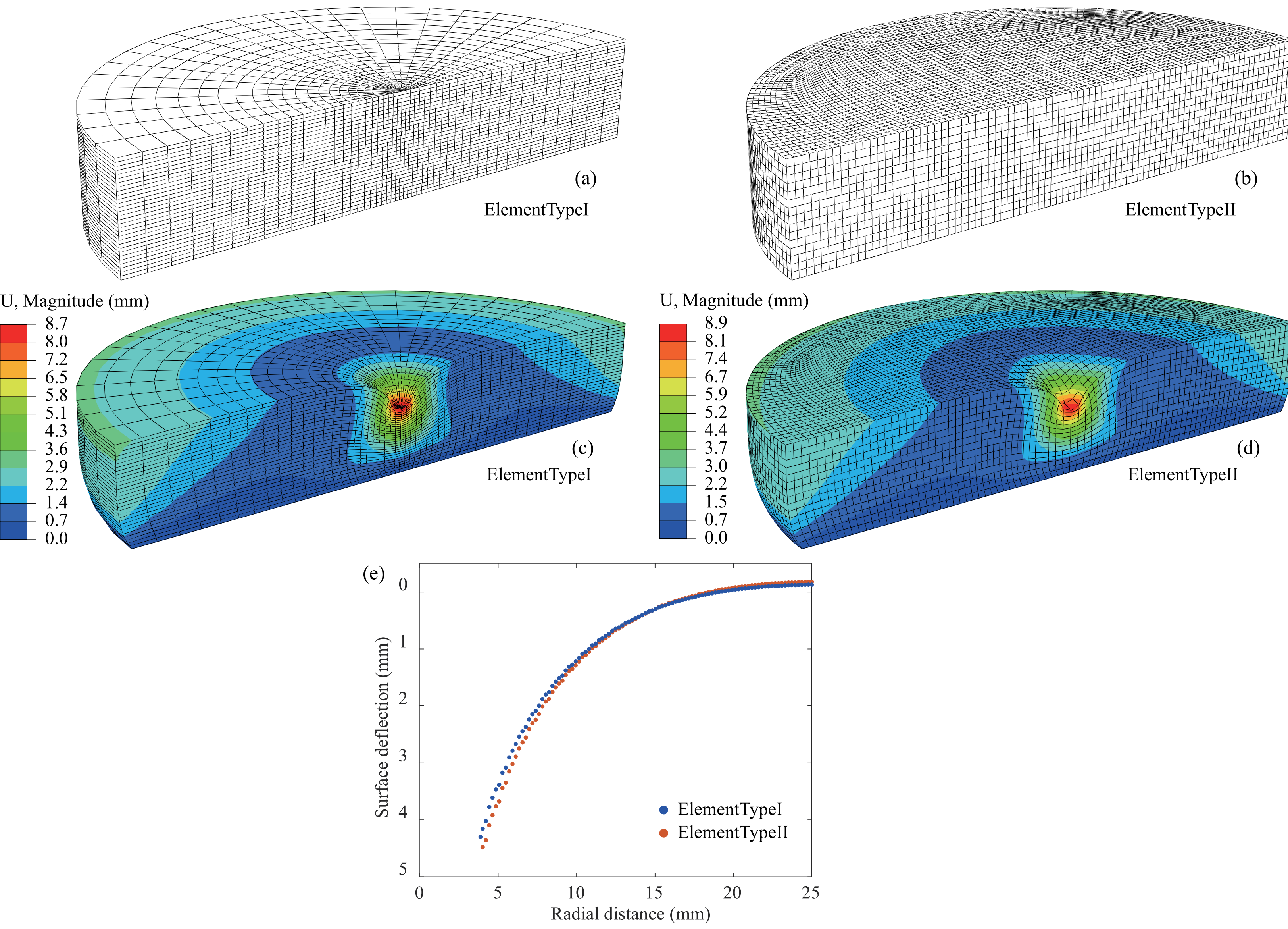

(a)
ElementTypeI
(b)
ElementTypeII
U, Magnitude (mm)
8.7
8.0
7.2
6.5
5.8
5.1
4.3
3.6
2.9
2.2
1.4
0.7
0.0
(c)
ElementTypeI
U, Magnitude (mm)
8.9
8.1
7.4
6.7
5.9
5.2
4.4
3.7
3.0
2.2
1.5
0.7
0.0
(d)
ElementTypeII
(e)
Surface deflection (mm)
0
1
2
3
4
5
Radial distance (mm)
0
5
10
15
20
25
ElementTypeI
ElementTypeII

Figure S2: *The FE models of the healthy tissue meshed with different element types and the corresponding modeling results. The FE model meshed with (a) element type I, (b) element type II; The modeling results of displacement distribution after indentation (c) with element type I, (d) with element type II; (e) Comparison of averaged indentation curves from the two models.*
